# Removal of Chromates and Sulphates by Mg/Fe LDH and Heterostructured LDH/Halloysite Materials: Efficiency, Selectivity, and Stability of Adsorbents in Single- and Multi-Element Systems

**DOI:** 10.3390/ma12091373

**Published:** 2019-04-28

**Authors:** Jakub Matusik, Karolina Rybka

**Affiliations:** Department of Mineralogy, Petrography and Geochemistry, Faculty of Geology, Geophysics and Environmental Protection, AGH University of Science and Technology, al. Mickiewicza 30, 30-059 Krakow, Poland; krybka@agh.edu.pl

**Keywords:** adsorption, halloysite, heterostructured adsorbent, magnesite, Mg/Fe LDH, wastewater

## Abstract

Industrial wastewaters often contain mobile and toxic anions that cannot be removed by precipitation techniques and most known adsorbents. Layered double hydroxides (LDH) are excellent scavengers of anions; however, their use in real applications is of minor importance owing to their swelling behavior and high cost of production. The performed research shows the possibility of obtaining Mg/Fe LDH using natural magnesite. Moreover, heterostructured LDH/halloysite materials were synthesized. The adsorption efficiency of these materials was very high in both single- and multi-element systems, confirming the LDH selectivity. This was with the exception of wastewaters containing a high concentration of chlorides, which clearly hampered the removal of Cr(VI) and S(VI). The measurements indicated that LDH dissolution took place to a small extent (<10 wt%). The LDH/halloysite materials showed lower efficiency than the raw LDH; however, the clay presence has several benefits in terms of future applications: (i) it significantly reduces the pH, especially in contrast to the calcined LDH, which enables the reuse or safe disposal of purified water; (ii) it reduces swelling of the composite, which opens the possibility for applications in column adsorption; (iii) it induces dual adsorption properties through additional cation adsorption; and (iv) it substantially lowers the price of the adsorbent.

## 1. Introduction

The continuous development of different industrial branches generates wastewaters of complex chemical composition, which subsequently increases the water pollution. So far, numerous methods have been designed for water treatment, and can be generally divided into three main groups: mechanical, chemical, and biological [1,2]. Among them, the use of adsorbents is relatively inexpensive and facilitates the removal of many types of pollutants. Adsorbents are receiving more attention because of their high effectiveness and wide range of applications both in industry and in environmental protection. Many materials have been designed for the removal of cationic heavy metals including nanoparticles, carbon-based materials (e.g., iron oxides, activated carbon, graphene oxides) [3,4], and clay minerals mostly of natural origin [5,6], as well as their hybrids [7,8,9]. The hybrids, in particular include clays and other solids, which play the role of supports for, as examples, magnetic nanoparticles, iron oxides, or zerovalent iron. Such an approach improves the stability and efficiency of the resulting hybrid composite. Many adsorbents have been reported as efficient materials for the removal of cations [10,11,12]; however, only some of them are capable of removing anions. Few reports confirmed the removal of anions by natural and modified zeolites [13,14], organo-kaolinite [15], modified montmorillonite [16], and modified halloysite [17,18,19].

Layered double hydroxides (LDH) are efficient anion exchangers, which are widely studied for the anionic species uptake from aqueous solutions [20]. LDH have a general formula, as follows: [M^II^_1-x_ M^III^_x_ OH_2_]^x+^ [A^n−^]_x/n_ y H_2_O, with divalent (M^II^ = e.g., Ca^2+^, Mg^2+^, Mn^2+^, Fe^2+^, Co^2+^, Ni^2+^, Cu^2+^, or Zn^2+^) and trivalent (M^III^ = e.g., Al^3+^, Fe^3+^) metals building brucite-like layers that are intercalated by anions (A^n−^ = e.g., Cl^−^, Br^−^, NO_3_^−^, CO_3_^2−^, SO_4_^2−^). The hydrated anions balance the positive charge of brucite-like layers, which result from M^II^/M^III^ isomorphous substitution. Owing to similarities of LDH when compared with natural clay minerals in terms of high surface area, as well as rheological and colloidal properties, these are often called anionic clays. Many natural types of LDH structures are known, including hydrotalcite (Mg–Al–CO_3_ LDH) and pyroaurite (Mg–Fe–CO_3_ LDH) [21]. Nevertheless, the LDH phases can be rarely found in nature, instead they can be easily synthesized in the laboratory.

One of the disadvantages of LDH synthesis is a relatively high cost of the necessary chemical reagents. Therefore, cheaper and widely available metal-bearing substrates are tested for their synthesis. Recently, a few minerals were used as sources of metals; for example, magnesite, dolomite, and kaolinite [22,23,24]. Moreover, in a few reports, LDH phases were synthesized using wastes, such as fly ash [25], furnace slag [26,27], aluminum wastes [28,29], and even brine water [30]. Because of the poor dispersity in water, which can affect LDH adsorption properties, some LDH hybrids have been synthesized in order to increase the available surface area and thus enhance adsorption capacity [31]. Several heterostructured materials consisting of LDH and other materials were reported, including nanocarbons [32], mesoporous silica [33], magnetite [34], sepiolite [35], and kaolinite [36,37]. Halloysite is a natural aluminum silicate of 1:1 layered structure belonging to the kaolin group of minerals, which exhibits various morphologies, such as plates and tubes. Halloysite can be an adequate support for the LDH to obtain a material with promising stability and adsorption capacity. Furthermore, there is a demand for studies regarding the stability of composites, and their affinity towards selected pollutants in multi-element systems, which can bring the research much closer to the practical applications on a larger scale [20,31].

Therefore, the goal of this study was to synthesize and characterize the less-studied Mg–Fe LDH from chemicals and magnesite, as well as LDH/halloysite heterostructured materials. The adsorption properties of these materials were tested towards hexavalent chromium Cr(VI) and sulphates S(VI) in model and multi-element solutions. In particular the study focused on examination of materials removal efficiency, selective adsorption properties and their stability in complex aqueous solutions.

## 2. Materials and Methods

### 2.1. Materials

For the synthesis, magnesite [M] from Szklary deposit (Lower Silesia, Poland) and halloysite-containing sample [H] from Dunino deposit (Lower Silesia, Poland) were used. The H sample is a kaolin rock containing ~60% of halloysite and ~40% of kaolinite, which was determined using a formamide test [38]. The sample also contained minor admixtures of crandalite and iron minerals (magnetite, ilmenite, and hematite). All chemical reagents used for the synthesis, including MgCl_2_·6H_2_O, FeCl_3_·6H_2_O, NaOH, and HCl, were of analytical grade. During the experiments, redistilled water was used.

### 2.2. Synthesis of LDH and H-LDH Composites

A total of eight materials were prepared: raw Mg/Fe LDH from chemical reagents (MgCl_2_·6H_2_O and FeCl_3_·6H_2_O) [LDH], Mg/Fe LDH from M and FeCl_3_·6H_2_O [LDHM], and their composites with H sample—[H-LDH] and [H-LDHM]. The molar ratio of Mg to Fe was set to 2:1, and the content of LDH to H sample was set to 25 wt%. Additionally, all materials were calcined at 450 °C for 3 h, which gave the following samples: [LDH-C], [LDHM-C], [H-LDH-C], and [H-LDHM-C].

The materials were synthesized by the co-precipitation method, after previous magnesite dissolution in pure HCl, in the case of LDHM and H-LDHM synthesis, in order to obtain Mg^2+^ solution. For the synthesis, solutions of 1.2 M Mg^2+^ and 0.6 M Fe^3+^ were prepared. Firstly, the pH of Mg^2+^ solution was set to 10 with NaOH. In the case of composites, a proper amount of H sample was dispersed in the Mg^2+^ solution. Secondly, the Fe^3+^ solution was added dropwise to the Mg^2+^ solution under continuous stirring with pH constantly controlled in the range of 9.0–10.0. Finally, the precipitate was aged for 24 h at room temperature (22 °C), then centrifuged (4500 rpm, 10 min), washed with redistilled water to eliminate the excess Na^+^, and dried at 60 °C overnight.

### 2.3. Adsorption Experiments in Single- and Multi-Element Solutions

The adsorption experiments for all eight materials were conducted both in single- and multi-element aqueous solutions, focusing on the changes of Cr(VI) and S(VI) concentrations. The Cr(VI) and S(VI) were introduced in the form of K_2_Cr_2_O_7_ and K_2_SO_4_, respectively.

In the single-element system, the 0.01 M NaCl solutions, with starting pH equal to 5.0, contained Cr(VI) or S(VI). The Cr(VI) initial concentration was set to 0.2, 1.0, and 5.0 mmol/L, while in the case of S(VI), it was equal to 1.0, 5.0, and 10.0 mmol/L. The used adsorbent dosage corresponded to 20 g/L.

The multi-element solutions were prepared from two wastewaters, CW (cooling water rich in Zn) and RW (rinsing bath water rich in chlorides), differing in chemical composition, which were obtained from a Polish metallurgical plant. Both of these wastewaters were spiked with Cr(VI) and S(VI) in amounts equal to 1.0 mmol Cr(VI)/L and 5.0 mmol S(VI)/L, in order to enable the results comparison with the single-element system. Finally, the four obtained types of multi-element solutions were prepared: Cr-CW, Cr-RW, S-CW, and S-RW. The Cr-CW and Cr-RW wastewaters contained Cr(VI), while S-CW and S-RW were spiked with S(VI). The initial pH was equal to 2.1 and the used adsorbent dosage was set to 20 and 40 g/L. For both the single- and multi-element systems, the adsorbents were shaken in appropriate aqueous solutions for 24 h. Afterwards, the samples were centrifuged.

The chemical composition of supernatants taken from single-element systems was analyzed using the 1,5-diphenylcarbazide colorimetric method for Cr(VI) and the BaCl_2_-based turbidimetric method for S(VI). In this case, the concentrations of Cr(VI) and S(VI) were calculated using the calibration curve approach, with absorption measured by UV-vis spectrophotometer (Hitachi U-1800 instrument, Tokyo, Japan). In turn, the full chemical composition of multi-element systems was determined using the ICP-OES method (Perkin Elmer Optima 7300 DV instrument, Waltham, MA, USA). All experiments were run in duplicates at room temperature. Adsorption efficiency was calculated from the difference between initial and equilibrium concentration of sorbate and was expressed in percentage in relation to initial concentration.

### 2.4. Analytical Methods

The obtained materials were characterized by X-ray diffraction (XRD), Fourier transformed infrared (FTIR) spectroscopy, scanning electron microscopy, and differential thermal analysis (DTA). The XRD patterns were obtained using a Miniflex 600 diffractometer with CuKα (λ = 1.5418 Å) radiation (Rigaku, Tokyo, Japan). The patterns of powdered samples were recorded in the range of 2–72°2θ with a 0.05°2θ step. The infrared spectra were collected by KBr pellet method (1 mg sample mixed with 200 mg KBr) with a Nicolet 6700 spectrometer (Thermo Scientific, Waltham, MA, USA). For each measurement, 64 scans were collected in the range of 4000–400 cm^−1^ and at a 4 cm^−1^ resolution. The SEM images were obtained using an Quanta 200 FEG microscope under low vacuum (FEI, Hillsboro, OR, USA). The samples were prepared by placing powdered material on a carbon tape. The thermal (DTA/TG) analysis of the samples was carried out using a STA 449F3 instrument coupled with a quadrupole mass spectrometer QMS 403 (Netzsch, Selb, Germany). The measurements were performed using ~20 mg samples in a temperature range of 25–1000 °C (heating rate: 10 °C/min, air atmosphere).

## 3. Results and Discussion

### 3.1. Characterization of Adsorbents

#### 3.1.1. XRD Results

**Figure 1:**
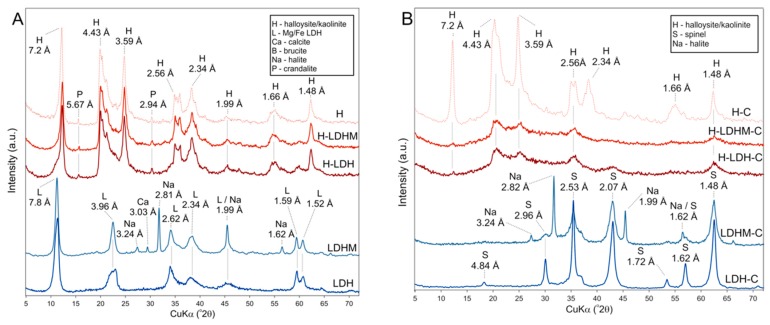
X-ray diffraction (XRD) patterns of (**A**) uncalcined and (**B**) calcined materials.

The XRD pattern of the LDH sample confirmed the presence of pyroaurite (ICDD #14-0293), which is a natural Mg–Fe LDH (Figure 1A). Characteristic peaks were found at 7.8, 3.96, 2.62, 2.34, 1.99, 1.59, and 1.52 Å, which refer to the d_003_, d_006_, d_012_, d_015_, d_018_, d_110_, and d_113_ values, respectively [39]. The basal reflection at 7.8 Å indirectly suggested the presence of CO_3_^2−^ as an interlayer anion in the Mg/Fe LDH [40,41]. The XRD pattern of LDHM sample, apart from the presence of pyroaurite, also showed reflections characteristic for halite and calcite. The first admixture was an effect of the reaction between NaOH and chloride reagents used for the synthesis. In turn, the latter calcite was present in the initial magnesite sample. In the case of H-LDH and H-LDHM composites, the halloysite reflections dominated, with its d_001_ value at 7.2 Å. However, the broadening of this reflection at ~7.8 Å can be noticed, confirming the presence of LDH. The LDH reflections were also visible as broadening or intensity increased in positions characteristic for the pyroaurite. The pattern also showed characteristic reflections for crandalite, which is a by-product of the H sample processing.

In the case of calcined samples, LDH-C and LDHM-C, the XRD pattern confirmed the formation of a spinel, magnesioferrite MgFe_2_O_4_, as a product of LDH sample calcination [42,43] (ICDD#01-1114) (Figure 1B). In analogy to the LDHM sample, as well as for LDHM-C, reflections characteristic for halite were denoted. The intensity of halloysite reflections was significantly lowered, or they were absent in the XRD pattern of the calcined composites, H-LDH-C and H-LDHM-C. This was because of the dehydroxylation of the halloysite-containing sample. The reflections at 2.96, 2.10, 1.71, 1.62, and 1.48 Å revealed the presence of magnesioferrite.

#### 3.1.2. FTIR Results

**Figure 2:**
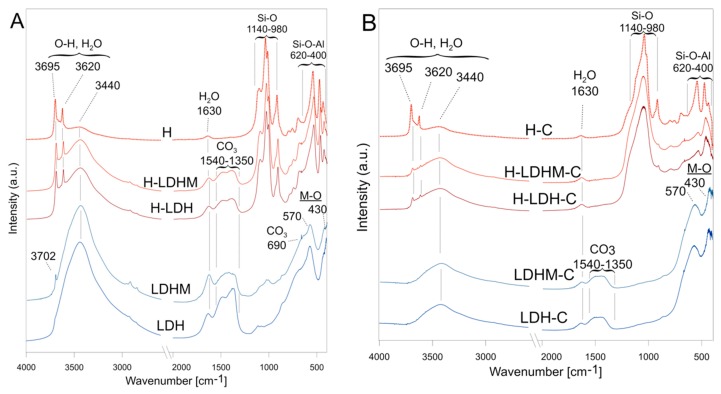
Fourier transformed infrared (FTIR) spectra of (**A**) uncalcined and (**B**) calcined materials.

The FTIR spectra of uncalcined samples showed bands connected to OH stretching and bending vibrations at 3440 cm^−1^ and 1630 cm^−1^, respectively, attributed to both surface bound and intercalated water molecules (Figure 2A). The intensity of these was much higher for the pure LDH and LDHM materials. The bands in the region of 1540–1350 cm^−1^ indicated the presence of intercalated CO_3_^2−^ anions, which was the result of CO_2_ adsorption from air during the synthesis as well as magnesite dissolution. In this region, two bands with maxima at 1480 and 1375 cm^−1^ were observed. These can be assigned to different carbonate species mostly of monodentate coordination [44]. The presence of bicarbonates can be excluded because of the lack of a 1220 cm^−1^ band [44]. Also, the band at 690 cm^−1^ was related to the presence of interlayer carbonates [45]. For the LDH and LDHM samples, the bands at 570 and 430 cm^−1^ were attributed to M–O–M and M–O stretching vibrations of the layered framework. Additionally, for the LDHM sample, a band at 3700 cm^−1^ represented the vibration of the Mg–O–H bond and indicated the formation of brucite [46]. The spectra of H-LDH and H-LDHM materials revealed bands at 3695 and 3620 cm^−1^, which were assigned to the stretching vibrations of inner surface and inner hydroxyls of the kaolin group minerals, respectively. The vibrations of aluminosilicate framework Si–O and Si–O–Al gave rise to the bands in the regions of 1140–980 and 620–400 cm^−1^.

The spectra of calcined samples (LDH-C and LDHM-C) showed a significant intensity decrease of bands attributed to OH groups and water, which confirmed LDH dehydroxylation (Figure 2B). The dehydroxylation of the structure and subsequent phase transformation associated with the release carbonates from the interlayer space resulted in the disappearance of the band at 690 cm^−1^. However, the spectra still showed bands (1540–1350 cm^−1^) attributed to CO_3_^2−^, which were formed as a result of CO_2_ adsorption from the air by magnesioferrite. In turn, the bands at 570 and 430 cm^−1^ were clearly visible, and were assigned to the lattice vibrations of magensioferrite [43].

The spectra of calcined composites (H-LDH-C and H-LDHM-C) showed evident changes in the regions of Si–O and Si–O–Al bonds due to kaolin group minerals dehydroxylation. Moreover, the intensity of bands at 3695, 3620, and 920 cm^−1^ attributed to the Al–O–H vibrations decreased. The spectra of heterostructured materials did not reveal the presence of CO_3_^2−^.

#### 3.1.3. SEM Results

**Figure 3:**
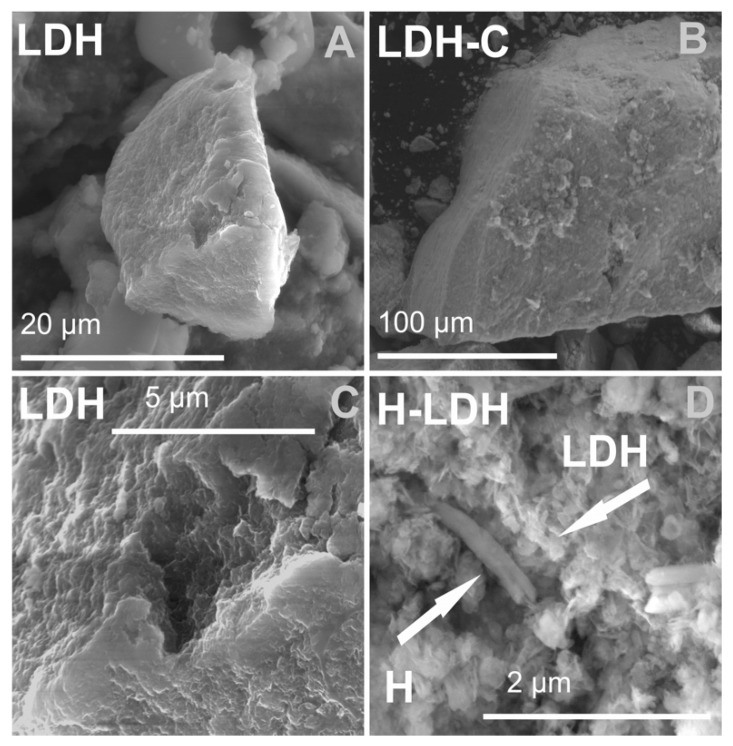
SEM images of the synthesized materials: (**A**,**C**) LDH, (**B**) LDH-C, and (**D**) H-LDH.

SEM images of the LDH and LDHM samples enabled the observation of a clearly visible layered morphology of the obtained materials, which reflected their internal structure (Figure 3A–C). For the H-LDH and H-LDHM samples, the LDH phase precipitated on the surface of H sample (Figure 3D), the particles of which showed a complex morphology: plates, tubes, and laths. The composite morphology clearly comprised two phases, namely micrometer sized halloysite particles and much smaller LDH platelets. The characteristic halloysite tubes visible in the images were on average 1–5 µm long with a diameter of ~0.2 µm. The stacked LDH plates of nanometer size very often surrounded halloysite tubes. The observations of calcined samples did not show significant changes of morphology when compared with the uncalcined analogues.

#### 3.1.4. Chemical Composition and DTA Results

Chemical analyses were performed in order to determine the Mg and Fe content for the LDH phases. The samples were dissolved in pure nitric acid, which was followed by measurement of Mg and Fe by atomic absorption spectroscopy (AAS) [47]. The results showed that the calculated Mg/Fe molar ratio was close to the assumed (2.0) value for the raw LDH materials: 2.06 ± 0.18 (LDH) and 2.13 ± 0.22 (LDHM). The Mg/Fe ratio in the case of heterostructured materials was found in the range of 2.08–2.33, which corresponded to the desired ratio of raw LDH materials.

**Figure 4:**
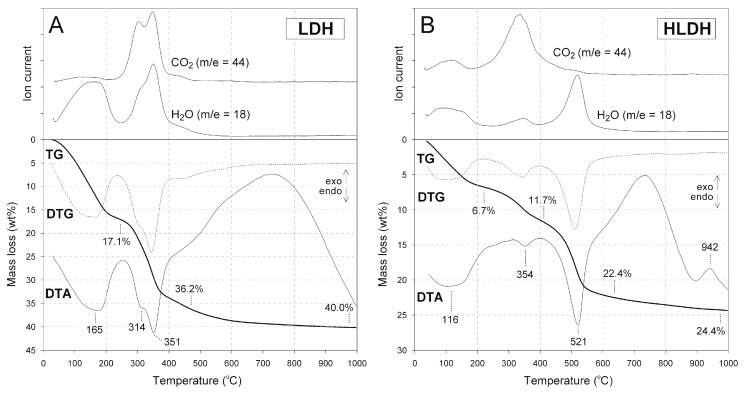
Thermal curves of (**A**) LDH and (**B**) H-LDH.

Thermal curves of the LDH and LDHM materials show similarities that confirmed that Mg/Fe LDH was formed during both syntheses (Figure 4A and Figure S1a), and stays in agreement with XRD and FTIR results. The observed decomposition stages were in agreement with earlier reports [48]. The first mass loss was attributed to release of water, which took place even up to 250 °C with maxima in the 140–160 °C range. This process was endothermic in character and was also confirmed by an appropriate H_2_O mass signal. Afterwards, these two samples underwent decarboxylation and dehydroxylation, which was evidenced by an endothermic effect in the range of ~250–500 °C. This effect showed two maxima related to the mentioned processes at around 310 °C and 345 °C, respectively. The mass spectra showed almost simultaneous start of CO_2_ and H_2_O release, which was characteristic for the carbonated LDH structure [49].

In the case of H-LDH and H-LDHM materials, the thermal effects connected to the LDH phase and kaolin group minerals were clearly distinct, which enabled their quantitative analysis (Figure 4B and Figure S1b). The dehydroxylation effect of the LDH took place at low temperatures, with the maximum at around 350 °C. In turn, dehydroxylation of H sample was evidenced by an effect with the maximum at 520 °C. The destruction of the brucite-like layer was accompanied by a release of both CO_2_ and H_2_O, while H sample decomposition released only H_2_O. This also helped to distinguish the mass losses attributed to both phases. The mass loss characteristic for H sample dehydroxylation equal to 10.7 wt% corresponded to the mineral content equal to 76.7 wt% by assuming standard decomposition of dehydrated aluminosilicate Al_2_Si_2_O_5_(OH)_4_ into Al_2_O_3_, 2SiO_2_, and 2H_2_O. Therefore, the LDH content of about 23.3 wt%, which was very close to the assumed 25.0 wt% content, was calculated for both of the heterostructured materials.

### 3.2. Adsorption in a Single Element System

**Figure 5:**
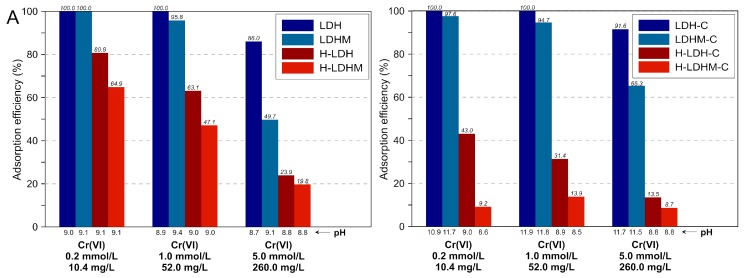

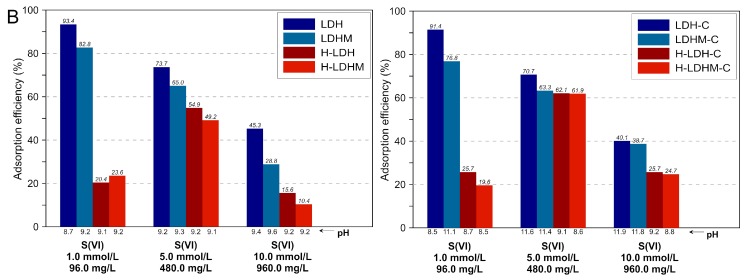
Adsorption efficiency of the materials in a single-element system: (**A**) Cr(VI) and (**B**) S(VI).

The experiments were designed to study the effect of the initial concentration (C_in_) of Cr(VI) and S(VI) on the removal efficiency by the synthesized materials. Figure 5A shows that the adsorption efficiency towards Cr(VI) decreased with increasing C_in_ for all the materials, reaching the lowest values equal to 19.8% for the H-LDHM and ~8.7% for the H-LDHM-C. The pure LDH phases were more efficient than composites with the H sample. However, it is worth to point out that the adsorption efficiency for the uncalcined composite with LDH prepared from magnesite (LDHM) was insignificantly lower than for the material with LDH prepared from chemicals (LDH). The adsorption efficiency for both the uncalcined and the calcined LDH and LDHM did not fall below 90% for C_in_ = 0.2 and 1.0 mmol/L. The uncalcined H-LDH and H-LDHM samples showed a good removal efficiency at low C_in_ of Cr(VI) equal to 80.8% and 64.9%, respectively. The affinity of calcined H-LDH-C and H-LDHM-C materials to Cr(VI) was much lower and was found below 43% for the lowest C_in_. The pH after adsorption (pH_eq_) for all materials increased in relation to initial pH (pH_in_) and varied between 8.7 and 9.4 for the uncalcined samples and between 8.5 and 11.8 for the calcined samples.

Adsorption efficiency towards S(VI), similarly to the efficiency towards Cr(VI), decreased with the increase in C_in_ (Figure 5B). Both the uncalcined and calcined materials showed a high removal efficiency for the lowest C_in_ found in the range of 76.8%−93.4%. For the highest C_in_, the removal was not observed to be lower than 28.8% for the LDHM sample. The composites with the H sample were less efficient in particular at higher C_in_, but the adsorption efficiency was not lowered below 10%. Similarly to Cr(VI), the pH_eq_ was higher in comparison with pH_in_ and varied between 8.7 and 9.6 for the uncalcined materials, and between 8.5 and 11.9 for the calcined materials.

### 3.3. Adsorption in a Multi-Element System

**Figure 6:**
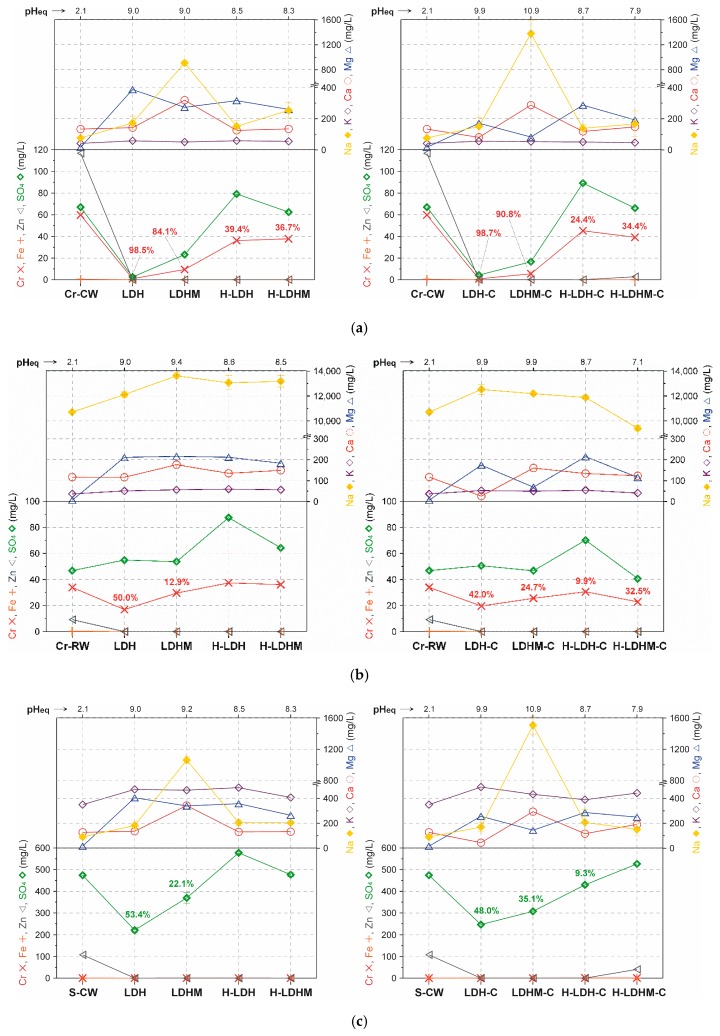

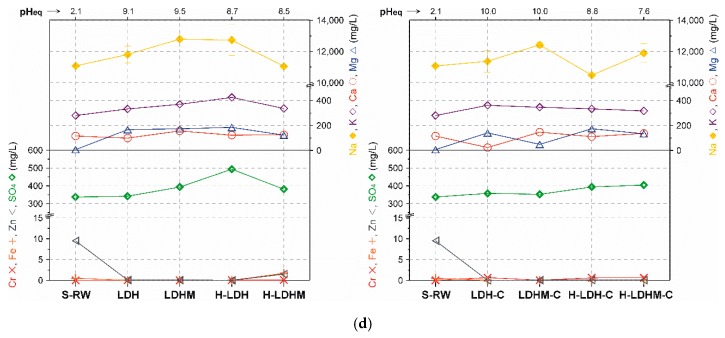
Equilibrium concentrations of selected elements (symbols given on y-axes) after adsorption experiments in multi-element wastewaters: (**a**) Cr-CW; (**b**) Cr-RW; (**c**) S-CW; (**d**) S-RW. The values on the graph indicate the percentage removal of Cr(VI) (Cr-CW and Cr-RW) or S(VI) (S-CW and S-RW).

The main objective of the experiments in multi-element systems was to investigate the selectivity of Cr(VI) or S(VI) removal by the studied materials and their stability in complex aqueous solutions. The chemical composition of used four wastewaters is shown in Table 1. In particular, the CW had a high concentration of Zn (>100 mg/L) and the RW was rich in Na and Cl (Na >10,000 mg/L, Cl >30,000 mg/L). These waters were spiked with Cr(VI) and S(VI) and their concentration was close to 1.0 mmol/L and 5.0 mmol/L, respectively. The choice of these concentrations enabled the comparison with results of the single element system. The removal of Cr, Fe, Zn, and SO_4_ as the main pollutants was investigated along with the analysis of Na, K, Ca, and Mg concentration changes (Figure 6). The Cr(VI) was below the detection limit in the waters spiked with S(VI)—S-CW and S-RW, where its C_in_ was low (Figure 6c,d). In turn, in the waters spiked with Cr(VI)—Cr-CW and Cr-RW, the adsorption efficiency of Cr(VI) significantly varied between 12.9% and 98.7% for the raw LDH phases and between 9.9% and 39.4% for the composites (Figure 6a,b). In the case of Cr-CW, the highest Cr(VI) uptake was observed for the LDH and LDH-C samples, equal to 98.5% and 98.7%, respectively (Figure 6a). The uptake was lower for the LDHM and LDHM-C samples; that is, 84.1% and 90.8%, respectively. For the both uncalcined and calcined composites, the Cr(VI) removal was in the 24.4% to 39.4% range. For the Cr-RW rich in chlorides, it was noticed that the Cr(VI) removal decreased significantly (Figure 6b). For the most efficient LDH and LDH-C materials, the uptake reached 50.0% and 42.0%, respectively. These values were lower for the LDHM and LDHM-C; that is, 12.9% and 24.7%, respectively. The uncalcined H-LDH and H-LDHM did not remove Cr(VI), while the adsorption efficiency for the calcined analogues was equal to 9.9% for H-LDH-C and 32.5% for H-LDHM-C.

The S(VI) were removed only from the S-CW, while their content was not lowered in the case of S-RW (Figure 6c,d). The LDH and LDH-C samples removed 53.4% and 48.0% of S(VI), respectively, from the S-CW. The LDHM and LDHM-C samples were less efficient and their efficiency was equal to 22.1% and 35.1%, respectively. The composites were also less efficient and only the H-LDH-C sample removed 9.3% of S(VI). Similarly to the Cr(VI) removal, the raw LDH phases were found to be more efficient than the composites.

Fe and Zn were removed completely by all the materials from all types of wastewaters. The K concentration did not change regardless of the type of water or material. Na and Ca were clearly released in the reactions with the LDHM material, which was an effect of the presence of halite and calcite, which underwent dissolution in the acidic conditions (pH_in_ = 2.1). This was particularly visible in the case of the RW waters, which were not rich in chlorides.

The Mg release and evolution of its concentration may be used as an indicator of LDH dissolution. The found Mg content was used to calculate the percent of dissolved LDH phase. For the LDH and LDHM samples, this percent in each of the four wastewaters was equal to Cr-CW (9.6% and 7.2%), Cr-RW (5.3% and 5.7%), S-CW (10.0% and 9.0%), and S-RW (4.1% and 4.6%). The analogical values for the calcined LDH-C and LDHM-C samples were as follows: Cr-CW (3.1% and 1.7%), Cr-RW (3.2% and 1.4%), S-CW (4.6% and 2.9%), and S-RW (2.5% and 1.0%). The latter set of values for the calcined materials indicated a very low release of Mg, which was in the structure of a spinel. In turn, the first set of values for the uncalcined materials showed a more pronounced release of Mg; however, in general, less than 10% of LDH was dissolved during the experiments. Moreover, it can be noticed that the high concentration of chlorides had a stabilizing effect on the uncalcined LDH structure, as in the case of RW, the dissolved percent of LDH was about two times lower. This is probably related to the aggregation of LDH and clay grains, which is enhanced in solutions with high concentrations of monovalent anions [50]. However, in this case, the adsorption was significantly hampered.

The Fe from the LDH dissolution was not detected after experiments and its absence was the result of instant precipitation at a higher pH_eq_, and most probably the formation of amorphous iron hydroxides. The presence of these phases was not confirmed as a result of very low C_in_ of Fe. In all cases, the pH_eq_ was higher for the raw LDH phases than for the composites, and even higher for the calcined materials.

The uptake of chlorides was not significant, however, it must be underlined that large amounts of chlorides clearly influenced the adsorption and were competitive with other anions. It was observed that the selectivity of the calcined materials was less affected by their presence. Similar observations were made for the removal of several oxyanions by different LDHs in seawaters with similar Cl^−^ concentrations [51,52,53]. The lower adsorption efficiency in RW wastewaters may have an effect of the aggregation of the adsorbent particles, which is enhanced in systems with a high concentrations of monovalent anions, and thus the decrease of specific surface area [50]. Beside chlorides, competition between sulphates and chromates was reported earlier [54], where the presence of chromates clearly influenced the removal of sulphates by Mg–Al LDH. Furthermore, chromates influenced the adsorption of cations. The increase of the Cr(VI) concentration lowered the removal of Zn and other metals by Mg–Al LDH [55].

**Figure 7:**
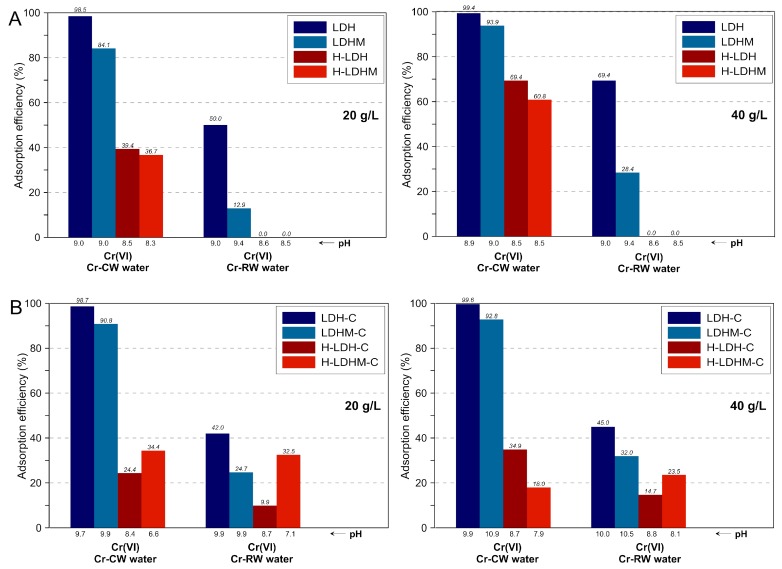
Comparison of Cr(VI) removal from multi-element systems versus adsorbent doses: (**A**) uncalcined materials and (**B**) calcined materials.

**Figure 8:**
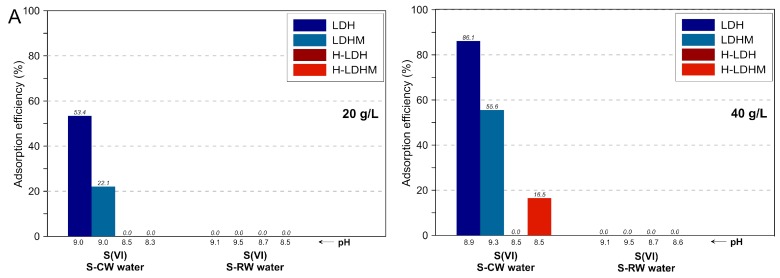

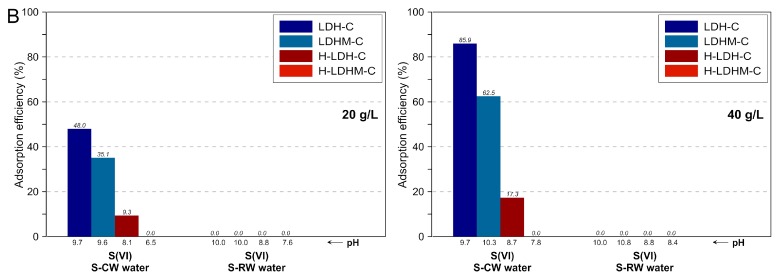
Comparison of S(VI) removal from multi-element systems versus adsorbent doses: (**A**) uncalcined materials and (**B**) calcined materials.

The effect of an adsorbent dosage increase from 20 to 40 g/L was studied for multi-element systems (Figure 7 and Figure 8). For the LDH and LDH-C samples, it was observed that the Cr(VI) removal efficiency from Cr-CW and Cr-RW remained at the same high level (Figure 7). In turn, the efficiency of these adsorbents increased in the case of S-CW, while the increase was not noticed for S-RW (Figure 8). For the magnesite-based LDHM and LDHM-C samples, a more significant increase of removal efficiency was noticed for the calcined sample in reaction with S-CW and S-RW. In the case of composites, a visible improvement was only observed for the uncalcined materials (H-LDH and H-LDHM) in reactions with the Cr-CW.

### 3.4. Solid State Analysis after Adsorption and Insight into Removal Mechanisms

**Figure 9:**
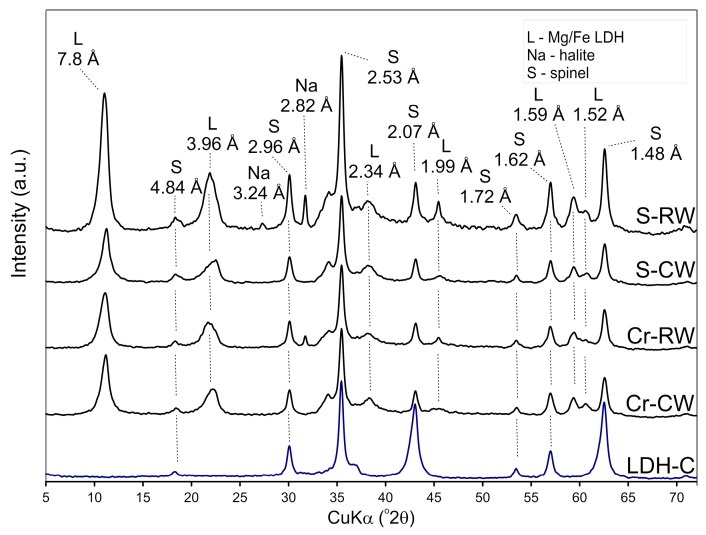
XRD patterns of the LDH-C material after adsorption in multi-element wastewaters.

In order to determine the mechanisms of the removal of anions, the samples, after adsorption experiments from the multi-element systems, were characterized by XRD and FTIR. The mechanism responsible for Cr(VI) and S(VI) uptake by the uncalcined materials was an anion exchange, while the memory effect involving the rehydration/reconstruction of LDH was responsible for anions’ uptake by the calcined materials [56]. The higher pH values observed for the latter were the result of the release of hydroxide ions during reconstruction of the LDH structure [31]. The XRD patterns of LDH sample after experiments with all waters did not reveal significant changes, with the exception of the appearance of a halite peak after the reaction with Cr-RW (Figure S2). This was an effect of NaCl precipitation from water rich in Na^+^ and Cl^−^. The changes of peak intensities and shape, in particular in the 20–25°2θ region, were the result of partial LDH dissolution, as attested above. The basal reflection was not shifted, which suggested that incorporation of Cr(VI) or S(VI) did not influence the interlayer space [41]. The memory effect was widely observed during the removal of anions from water solutions by calcined LDH [20]. It was noticed that the LDH-C sample after adsorption experiments was not completely rebuilt, although the 7.8 Å basal reflection appeared after the reactions (Figure 9). This was because the XRD pattern revealed both the peaks characteristic for pyroaurite (L—Mg/Fe LDH) and MgFe_2_O_4_ (S—spinel), which was the product of LDH calcination. In the samples after the experiment with RW, the presence of halite was also confirmed. The calcined composites H-LDH-C and H-LDHM-C did not show any structural changes after the experiments (data not shown).

**Figure 10:**
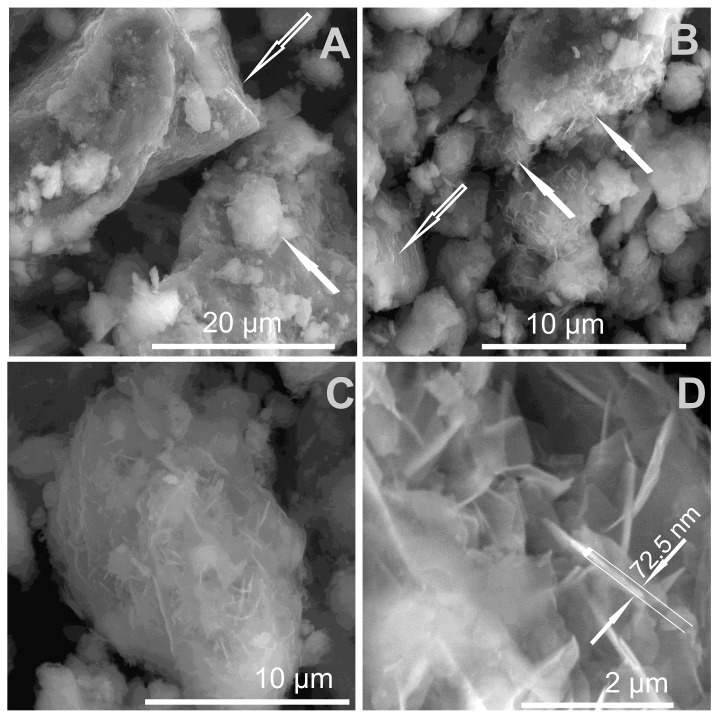
SEM images of the calcined materials after adsorption experiments (**A**–**D**). The arrows in (**A**,**B**) indicate newly formed LDH.

The reconstruction of LDH-C was also confirmed by FTIR spectra. The 570 and 430 cm^−1^ bandswere unaffected by calcination while the band at 690 cm^−1^ reappeared as a result of the incorporation of CO_3_^2−^ (Figure S3). Moreover, the bands attributed to intercalation of carbonates were visible in the range of 1540–1350 cm^−1^. The SEM observations indicated morphological differences between treated uncalcined and calcined phases. The first did not show morphology changes, however, in the case of calcined materials, newly formed clusters built from LDH plates were often observed (Figure 10). These agglomerates had a total diameter in the range of 5–20 µm. Within the clusters, the LDH plates forming characteristic “house of cards” structures were observed. The individual particles of LDH had thicknesses in the range of 50–80 nm (Figure 10).

**Figure 11:**
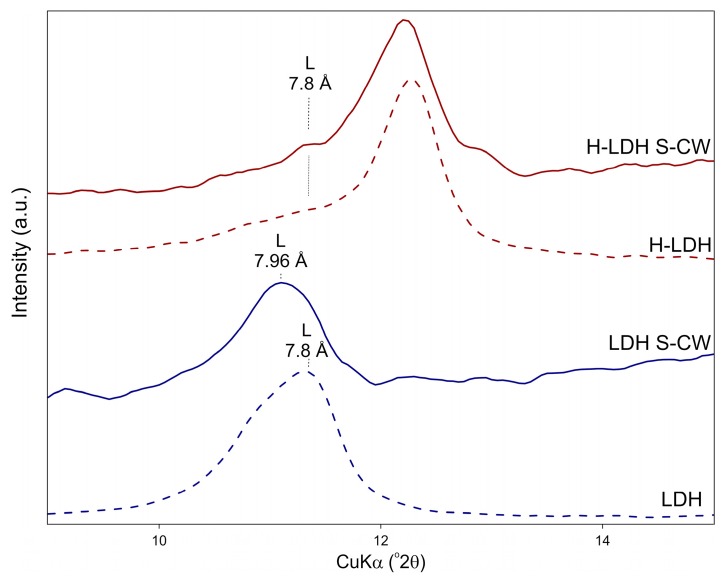
XRD patterns of LDH and H-LDH after reaction with S-RW (recorded in a wet state).

In order to investigate the swelling behavior of the obtained materials in aqueous solutions, the XRD analysis of wet samples was performed. The materials were analyzed immediately after adsorption experiments in multi-element systems. For the LDH sample, a basal peak shift from 7.8 Å to 7.96 Å was noticed (Figure 11). While for the H-LDH, the position of the LDH basal reflection remained the same, indicating reduced swelling properties of halloysite-containing composites. This is an advantage of the composites in terms of possible future applications in dynamic adsorption systems.

## 4. Conclusions

The performed research showed the possibility of obtaining a Mg/Fe LDH material using natural magnesite sample as a source of Mg^2+^. Furthermore, the heterostructured materials of LDH and halloysite-containing sample (25:75 mass ratio) were prepared by precipitation of LDH directly in the clay dispersion. All the samples were tested in their uncalcined and calcined versions in single- and multi-element aqueous solutions spiked with Cr(VI) or S(VI). The Mg/Fe LDH synthesized from chemicals had very similar structural features and adsorption efficiency as compared with the magnesite-based LDH. This was attested both in single-element and multi-element solutions, which confirmed excellent selectivity of LDH towards Cr(VI) and S(VI) anions. This is worth underlining as a mineral-based synthesis approach to adsorbents may significantly reduce the costs, mainly connected with necessary chemical compounds. For all materials, the experiments showed that a very high concentration of chlorides (RW waters) hampered the removal of Cr(VI) and S(VI) to a great extent, and thus efficient purification was not possible.

The LDH/halloysite composites in general were less efficient towards selected elements than the LDH phases, however, important benefits of such heterostructured materials must be emphasized in context of future applications. Because of the high availability and low price of halloysite, the clay material will heavily reduce the overall adsorbent price. Thus, if necessary, higher adsorbent doses may be used for water purification, as the results showed that the increase of dose from 20 to 40 g/L improved Cr(VI) removal by the composites in water of low ionic strength (Cr-CW). The use of halloysite as a clay component in the composite mass reduces the final pH of treated waters by one to two orders of magnitude, which facilitates their disposal or further reuse in industrial processes. In contrast, the pH increased significantly when raw LDH was used, especially in the case of calcined materials. The measurements clearly showed that in the applied experimental conditions, the LDH dissolution and simultaneous release of Mg^2+^ takes place to a small extent. This effect will be further limited in the case of composites where LDH content should not be higher than 25 wt%. The halloysite presence will undoubtedly facilitate the preparation of granulated adsorbents and reduce swelling of LDH in dynamic adsorption flow-through systems, as evidenced in this work. It is particularly important as the LDH swelling is one of the reasons why pure LDH are not used in industrial applications for adsorption [57].

## Figures and Tables

**Table 1:** Chemical composition and pH of Cr-CW, Cr-RW, S-CW and S-RW wastewaters.

mg/L	Cr-CW	Cr-RW	S-CW	S-RW
Cr(VI)	59.7	33.9	0.09	0.01
S(VI)	66.9	46.8	474.1	337.5
Cl^−^	750.0	>30,000	750.0	>30,000
Na^+^	76.7	10,710	89.9	11,072
K^+^	41.6	36.8	349.2	280.0
Ca^2+^	133.7	117.5	127.4	115.8
Mg^2+^	16.7	6.9	16.4	6.7
Fe_total_	0.33	0.23	0.21	0.49
Zn^2+^	116.7	9.21	108.1	9.52
pH	2.1	2.1	2.1	2.1

## Supplementary Materials

The following are available online at https://www.mdpi.com/1996-1944/12/9/1373/s1, Figure S1: Thermal curves of (a) LDHM and (b) H-LDHM; Figure S2: XRD patterns of the LDH material after adsorption in multi-element wastewaters; Figure S3: FTIR spectra of the LDH material after adsorption in multi-element wastewaters.
