# Supplementary material for: Removal of Chromates and Sulphates by Mg/Fe LDH and Heterostructured LDH/Halloysite Materials: Efficiency, Selectivity, and Stability of Adsorbents in Single- and Multi-Element Systems
Source: Materials (Basel). 2019 Apr 28;12(9):1373. doi: 10.3390/ma12091373 (PMC6539779; doi:10.3390/ma12091373)
Supplement: Supplementary file 1 [file materials-12-01373-s001.pdf]

materials
MDPI

Supplementary Information

Removal of Chromates and Sulphates by Mg/Fe LDH and Heterostructured LDH/Halloysite Materials: Efficiency, Selectivity, and Stability of Adsorbents in Single- and Multi-Element Systems

Jakub Matusik * and Karolina Rybka

Department of Mineralogy, Petrography and Geochemistry, Faculty of Geology, Geophysics and Environmental Protection, AGH University of Science and Technology, al. Mickiewicza 30, 30-059 Krakow, Poland; krybka@agh.edu.pl

* Correspondence: jmatusik@agh.edu.pl; Tel.: +48-126175233

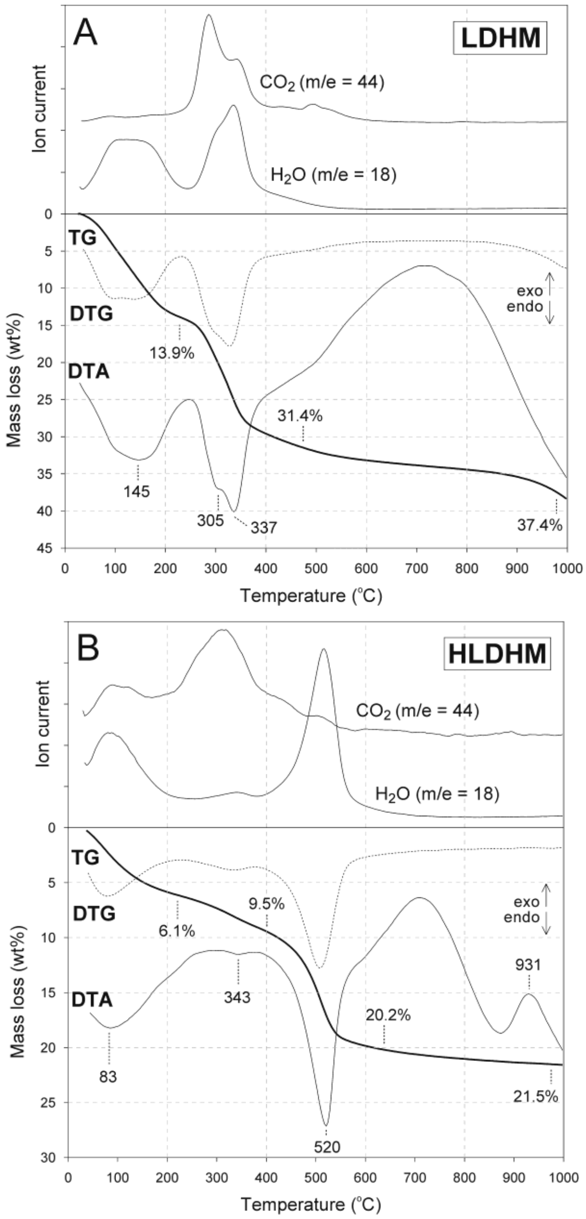

Figure S1. Thermal curves of: (**a**) LDHM; (**b**) H-LDHM.

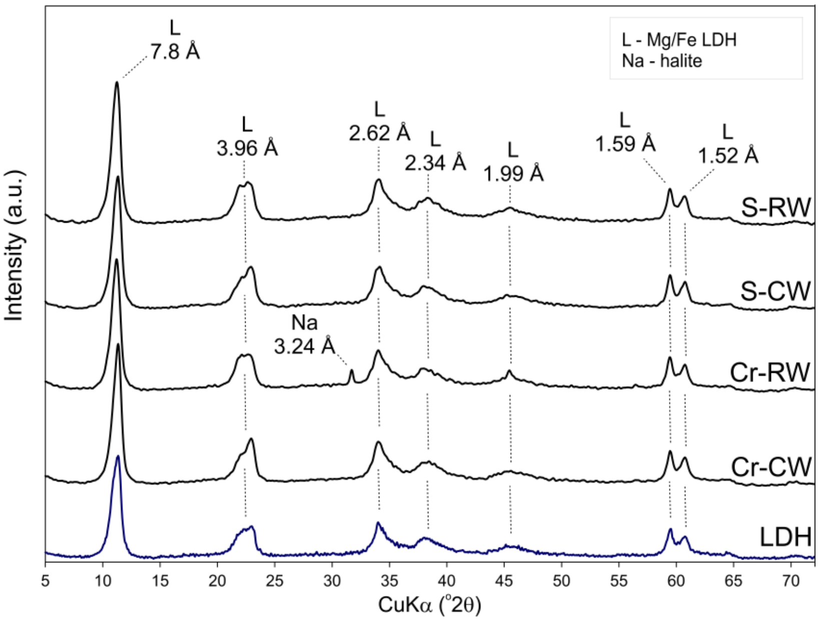

Figure S2. XRD patterns of the LDH material after adsorption in multi-element wastewaters.

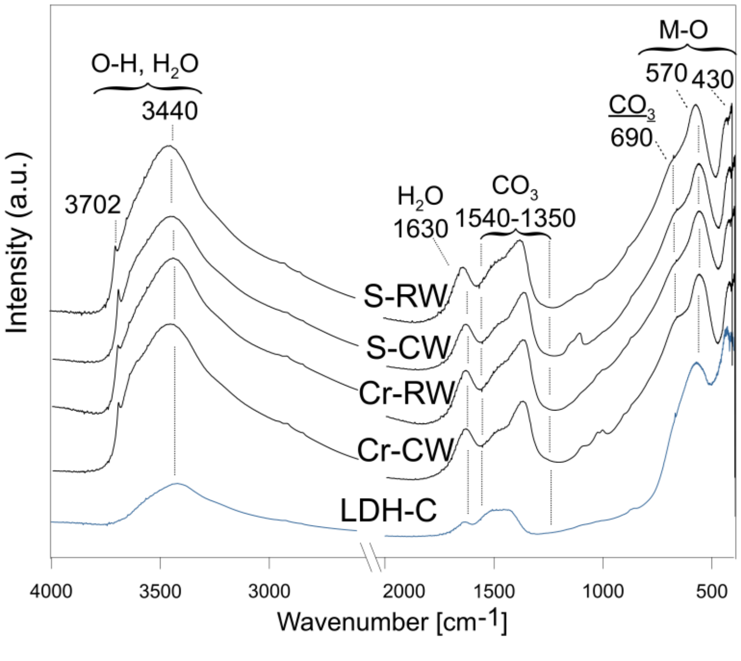

Figure S3. FTIR spectra of the LDH material after adsorption in multi-element wastewaters.
